# Pain, cognition, and culture in dementia research: a critical synthesis of empirical and methodological literature

**DOI:** 10.3389/fpain.2026.1797629

**Published:** 2026-10-09

**Authors:** Rimsha Taqdees, Tom Mercer, Abigail Olubola Taiwo

**Affiliations:** 1 Faculty of Science and Engineering, School of Psychology and Sport, University of Wolverhampton, Wolverhampton, United Kingdom; 2 Department of Psychology and Sports, School of Health, Medicine and Life Sciences, University of Hertfordshire, Hatfield, United Kingdom

**Keywords:** cognitive decline, culture, dementia, pain, pain assessment

## Abstract

Research examining relationships between pain and cognition in dementia and cognitive impairment has expanded, yet methodological and cultural limitations remain insufficiently explored. This paper critically synthesises findings from two scoping reviews focusing on pain-cognition relationships in dementia, methodological approaches used in assessing these constructs, and the influence of cultural factors on pain and cognition. Across the included reviews, evidence suggests chronic pain was generally associated with poorer cognitive performance, particularly in domains such as executive functioning and processing speed, although findings varied across studies. This variation may partly reflect differences in measurement tools and study design, particularly the use of global screening tools versus domain-specific cognitive assessments. The synthesis indicates that interpretation of evidence in this area is shaped by inconsistent cognitive assessment practices and reliance on pain measures that become less appropriate with cognitive decline. The review argues that culturally informed and multimodal approaches are required to improve assessment validity and advance dementia research and care.

## 1 Introduction

Pain is a common yet frequently under-recognised symptom in dementia, with growing evidence suggesting that it may influence cognitive functioning (1–4) Evidence suggests that pain is not only highly prevalent in people living with dementia but is also closely linked to neuropsychiatric symptoms. For example, Chejor et al. found no significant differences in pain prevalence between immigrant and non-immigrant residents in aged care homes; however, non-English-speaking immigrants exhibited significantly higher pain intensity scores (5). Total pain severity was strongly associated with neuropsychiatric symptom burden across all groups, highlighting an association between pain severity and neuropsychiatric symptoms in dementia, and suggesting that cultural and linguistic factors may influence how pain is expressed and potentially recognised in care settings. Similarly, the recognition and assessment of pain in dementia can be complicated by difficulties in communication, reduced self-awareness, and progressive cognitive decline, increasing the risk of under-treatment and poorer quality of care (6–9) Recent evidence suggests that chronic pain is associated with an increased risk of cognitive impairment, particularly dementia, with pooled estimates indicating a modest but consistent elevation in risk, although findings vary across study designs and follow-up durations (10).

Conversely, cognitive impairment may alter the way pain is perceived, expressed, and managed (11, 12), suggesting a complex and potentially bi-directional relationship between pain and cognition. The association between chronic pain and cognitive performance has increasingly been explored in existing literature, particularly in relation to executive functioning, attention, memory, and processing speed (13–18) Studies have suggested that chronic pain may contribute to poorer cognitive outcomes, while others indicate that cognitive impairment itself may interfere with accurate pain reporting and management (19–22) Despite growing interest in this area, the findings remain inconsistent due to substantial methodological variation across studies including differences in pain assessment strategies, cognitive measures, participant populations, and subtypes of dementia (23, 24). The current evidence base remains fragmented, limiting coherent interpretation across studies. Chronic or unmanaged pain may contribute to poorer cognitive functioning through mechanisms such as attentional interference and reduced engagement in daily activities, while cognitive impairment may interfere with the recognition, communication, and reporting of pain (25–28)

Alongside conceptual limitations, there are important methodological challenges that are associated with assessing both pain and cognition in dementia populations. Self-report pain measures, although widely regarded as the gold standard in pain assessment, become increasingly difficult to administer reliably as cognitive impairment progresses (29–31) Observational measures offer an alternative approach (32, 33) but are also subject to limitations including observer interpretation and variability in behavioural presentation (34–36) Recent evidence suggests that although dementia-specific pain assessment tools such as Pain Assessment in Advanced Dementia (PAINAD) are widely used in emergency department settings, there is still no clear evidence that these tools improve timeliness or effectiveness of analgesia delivery in comparison to standard pain scales (37). This highlights persistent uncertainty in the effectiveness of pain assessment strategies for people living with dementia in acute care environments. Commonly used cognitive screening tools similarly provide broad indications of cognitive functioning but may lack sensitivity to more specific cognitive domains that are affected by pain (38, 39). This distinction between dementia risk and continuous cognitive performance measures further highlights uncertainty regarding how pain relates to different aspects of cognitive functioning, particularly when different cognitive outcomes are assessed using different measurement approaches (10).

Cultural influences remain underexplored in dementia research on pain and cognition. The expression of pain, its communication, coping strategies, and help-seeking behaviours are shaped by cultural norms and values (40–42) Standard neuropsychological assessment in Arabic-speaking populations may be influenced by diglossia, literacy levels, and translation issues, which can contribute to misinterpretation or over/under-diagnosis of cognitive impairment (43), yet many commonly used assessment tools are developed and validated within primarily Western populations.

Similarly, cognitive assessment may not fully account for cultural and linguistic differences that influence test performance (44–46) Standardised neuropsychological assessments may therefore be influenced by differences in language proficiency, educational background, and cultural familiarity with test materials which can contribute to misleading interpretations of cognitive performance in migrant populations (47). Beyond assessment challenges, evidence concerning dementia care for Arabic-speaking migrants and refugees remains limited, with no clear consensus on culturally and linguistically appropriate neurocognitive screening, assessment, and care (48). Language interpretation may support communication during healthcare and assessment encounters (49); however, it does not necessarily address the cultural and linguistic validity of the assessment tools themselves, particularly where performance may be influenced by translation, literacy, language structure, educational background, or cultural familiarity with test materials (50). Challenges relating to linguistic variation, literacy, translation, and cultural familiarity with assessment materials may still influence cognitive assessment performance and interpretation. The availability of interpretation therefore does not necessarily resolve the lack of consensus regarding culturally and linguistically appropriate neurocognitive screening and assessment across diverse Arabic-speaking populations.

Cultural and linguistic factors further complicate the assessment of pain in dementia populations. This is supported by evidence from Chejor et al. who found immigrant status and language proficiency may influence observed pain intensity (5). This suggests that while pain occurs at comparable rates across groups, its expression or recognition may differ depending on cultural and language context, reinforcing concerns about inequities in assessment and interpretation of pain in multicultural dementia populations. The limited integration of cultural factors raises concerns about the generalisability of findings and the cultural validity of commonly used assessment approaches.

The limited evidence base concerning dementia care for Arabic-speaking migrant and refugee populations, together with the lack of consensus regarding culturally and linguistically appropriate screening, assessment, and care, underscores the need for more inclusive approaches to dementia-related health research (48).

Given these limitations, there is a need to critically examine not only the empirical relationship between pain and cognition in dementia, but also the methodological approaches used to assess these constructs. This paper presents a critical synthesis of two scoping reviews that were conducted as part of a doctoral research programme. The first review explored empirical relationships between pain and cognition in dementia and cognitive impairment, while the second examined methodological approaches used in pain and cognitive assessment within these populations. By synthesising findings from both reviews, this paper aims to explore the three interrelated areas of the relationship between pain and cognition in dementia, the methodological approaches used to assess these constructs, and cultural influences on the assessment and interpretation of pain and cognition. Ultimately, the synthesis seeks to contribute towards the development of more inclusive and methodologically robust approaches to dementia research and care.

## 2 Methodology

### 2.1 Design

This paper presents a critical analysis of two scoping reviews conducted as part of a doctoral research programme examining pain, cognition, and cultural influences in dementia. A critical synthesis was used to enable the integration of empirical findings and methodological issues identified across the two reviews, while also facilitating a broader conceptual examination of gaps within the literature.

Although systematic reviews encompass a diverse range of approaches to synthesising evidence, a critical synthesis enables the interpretive integration of heterogeneous findings across diverse conceptual and methodological domains (51, 52).

The synthesis was guided by an interpretive and comparative analytical process, drawing together convergences and divergences across the two reviews in order to identify broader themes relating to pain assessment, cognitive measurement, methodological limitations, and cultural exclusion within dementia research.

### 2.2 Eligibility criteria

Studies were included if they involved human participants with dementia or cognitive impairment, or chronic pain conditions; if they examined pain, cognition, or related assessment practices; or if they used experimental, observational, or descriptive designs. Both reviews were originally conducted in 2021 and restricted inclusion to English-language articles published during the preceding five years, in order to capture contemporary methodological developments and perspectives available at the time of the searches.

Although the two reviews addressed distinct research questions, they used broadly similar eligibility criteria and search methods because both examined pain, cognition, and related assessment practices in dementia and cognitive impairment. To avoid unnecessary duplication, the shared criteria and search procedures are described together below, while the review-specific aims and methodological differences are described separately in Section 2.4.

### 2.3 Search strategy

Literature searches were conducted in PubMed and PsycINFO in 2021 using combinations of search terms relating to pain, cognition, dementia, communication, and cultural factors. PubMed and PsycINFO were selected because together they provided complementary coverage of biomedical, health, psychological, and cognitive research directly relevant to the review questions. The five-year publication limit was applied in relation to the timing of the original searches, which were conducted in 2021, in order to capture contemporary methodological developments and perspectives available at that time. A broadly consistent search strategy was used across both reviews to ensure that the complementary reviews examined a comparable body of literature and to support consistency in the subsequent synthesis. Search terms were combined using Boolean operators to capture variations in terminology across the literature. The full search strategy, including all keyword combinations and results, is provided in Supplementary Material A.

Figures 1,2 present the study selection process for the two scoping reviews included in the synthesis.

**Figure 1:**
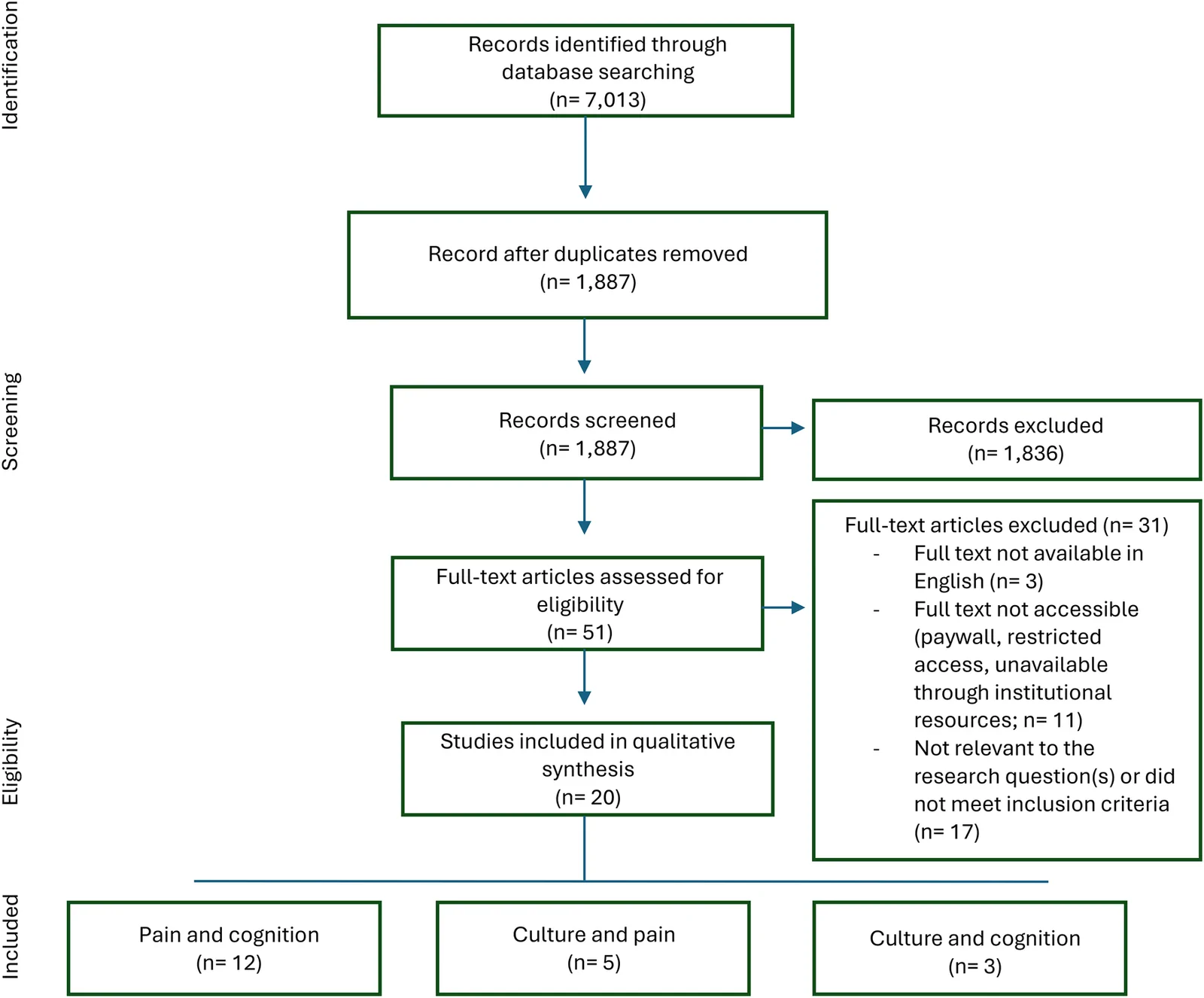
**Note:** PRISMA flow diagram for scoping review 1.

**Figure 2:**
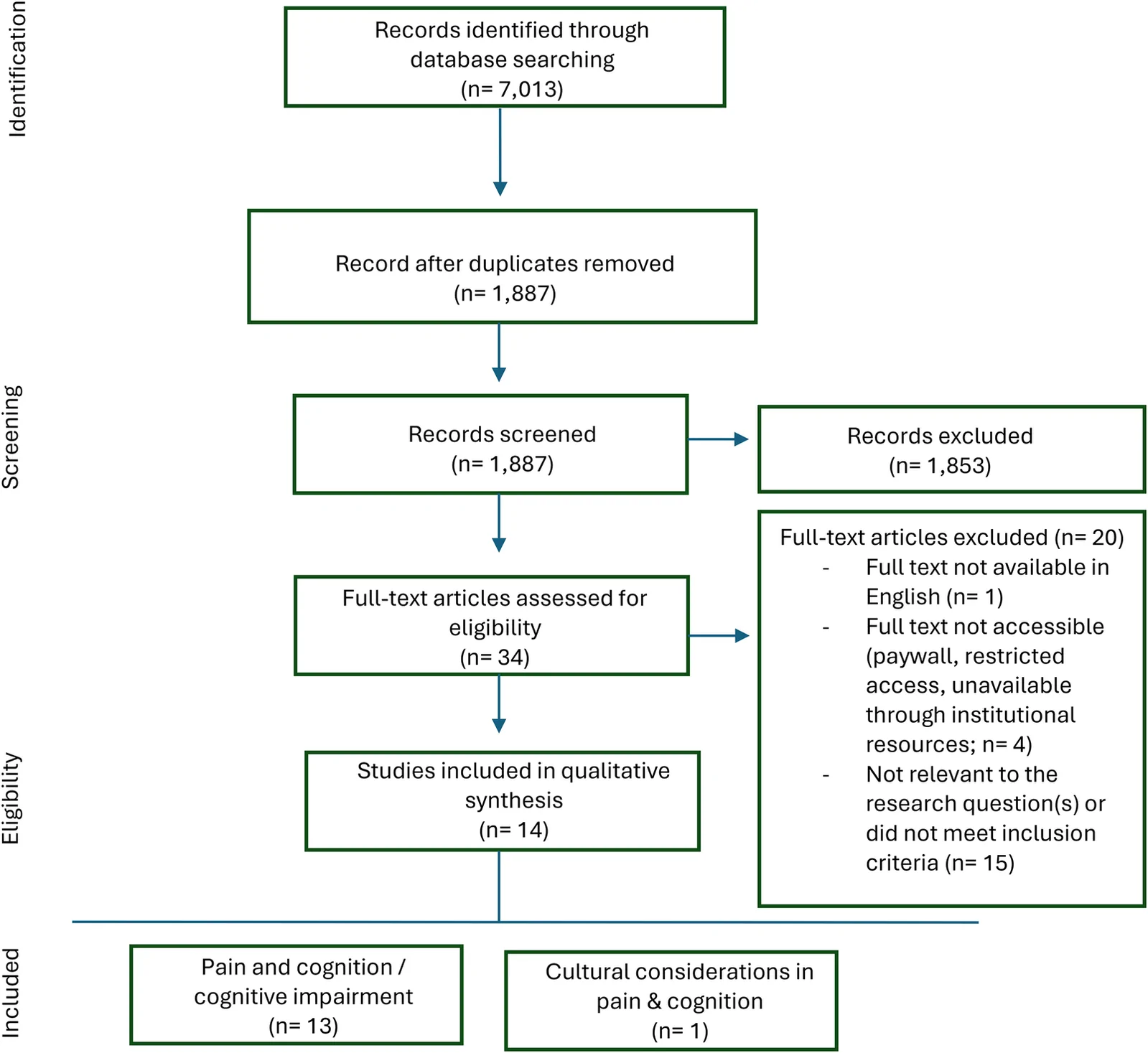
Flowchart illustrating a scoping review process: 7,013 records identified, 1,887 after duplicates removed, 1,887 screened, 1,853 excluded, 34 full-text articles assessed, 20 excluded for various reasons, 14 studies included, with 13 on pain and cognition or cognitive impairment, and 1 on cultural considerations in pain and cognition.

### 2.4 Included reviews

The synthesis integrated findings from two scoping reviews conducted by the author during doctoral research. Patterns identified within the extraction tables informed the thematic and interpretive synthesis presented within the results and discussion sections.

Review 1: Bridging the Gaps: Pain, Cognition, and Culture in Dementia Care

The first scoping review explored empirical associations between pain and cognition in individuals with dementia and cognitive impairment. The review was conducted in line with the Joanna Briggs Institute (JBI) methodology for scoping reviews (53).

This review included studies examining pain, cognition, dementia, and cultural influences across clinical and care settings. Both experimental and observational study designs were eligible. Data extraction included study characteristics, participant populations, assessment approaches, and key findings related to pain and cognition.

Review 2: Assessing Pain and Cognitive Performance in Dementia and Chronic Pain: A Scoping Review of Existing Methods and Gaps

The second scoping review examined methodological approaches used to assess pain, cognitive performance, and cultural variables within dementia and cognitive impairment research. The review also followed JBI guidance for scoping reviews (53).

The review explored methodological practices relating to pain assessment tools, cognitive measures, and cultural variables across dementia and chronic pain populations. Included studies comprised experimental, observational, and descriptive designs. Data extraction focused specifically on assessment tools, methodological strengths and limitations, and gaps within current approaches.

### 2.5 Rationale

The two reviews were synthesised due to their complementary focus areas. While the first review examined empirical relationships between pain and cognition in dementia and cognitive impairment, the second focused on methodological approaches used to assess these constructs. Synthesising these reviews enabled both conceptual findings and methodological limitations within the literature to be examined simultaneously.

This combined approach allowed for a broader critical evaluation of how pain and cognition are currently conceptualised and measured within dementia research, while also identifying limitations related to cultural representation, assessment, validity, and methodological consistency. The synthesis therefore extended beyond a descriptive summary of findings and aimed to generate a more integrated understanding of gaps within the existing evidence base.

### 2.6 Analysis

Findings from both reviews were analysed using a comparative and interpretive approach. Key findings, methodological characteristics, and identified research gaps from each review were compared to identify areas of convergence, divergence, and recurring patterns across the evidence base. Particular attention was given to relationships between pain and cognitive functioning, methodological approaches to pain and cognitive assessment, limitations of self-report and observational measures, variability in cognitive assessment practices, and the consideration of cultural influences within dementia research.

Areas of convergence and divergence across the reviews were identified and interpreted critically to develop a broader understanding of methodological and conceptual limitations within the field. The synthesis process focused not only on summarising findings, but also on examining how methodological approaches to the assessment of pain and cognition may influence the interpretation of reported relationships.

Data were first charted within structured extraction tables and then compared across studies to identify recurring patterns in assessment approaches, reported outcomes, and research gaps. These patterns informed the organisation of the results and the critical discussion presented in subsequent sections. Detailed extraction tables for both scoping reviews are provided in Supplementary File B.

Although formal quality appraisal was not undertaken, consistent with scoping review methodology, methodological limitations of included studies were considered during the interpretive synthesis and discussion.

## 3 Results

### 3.1 Overview of studies included in scoping review 1

In scoping review 1, 20 studies were included in the synthesis examining associations between pain, cognition, and dementia. The included studies comprised a range of methodological designs, including cohort studies, cross-sectional analyses, qualitative studies, systematic reviews, experimental studies, and intervention-based research.

Of the 20 studies included in scoping review 1, 5 were cohort/longitudinal (4, 54–57), 3 cross-sectional (58–60), 2 qualitative (61, 62), 3 systematic reviews (22, 63, 64), 2 experimental (34, 65), 2 intervention studies (66, 67), 1 narrative review (68), 1 mixed-methods dissertation (69), 1 additional review/theoretical synthesis (70).

The evidence base was predominantly drawn from older adult populations, including individuals with dementia, nursing home residents, and community-dwelling older adults. Clinical subgroups (e.g., post-surgical or hospitalised older adults with cognitive impairment) were also represented.

Across the included studies, pain assessment approaches varied and included self-report measures, observational tools, facial expression-based methods, and mixed or multimodal approaches. Cognitive outcomes were assessed using a combination of global screening tools, such as the MMSE and MoCA, and domain-specific neuropsychological measures, including tests of executive functioning, memory, attention, and processing speed. Some studies used more than one assessment approach; therefore, these categories are not mutually exclusive.

The included studies demonstrate considerable methodological heterogeneity in both pain and cognitive assessment approaches, with variation in study design, measurement tools, and population characteristics. Detailed study characteristics, including extraction of methodological variables and assessment tools, are provided in Supplementary File B.

### 3.2 Empirical findings on pain-cognition relationships

Across the included studies, both chronic and persistent pain were frequently associated with poorer cognitive outcomes in older adults and dementia populations. Meta-analytic and cohort evidence indicated that pain is associated with accelerated cognitive decline, particularly in domains such as processing speed, executive functioning, and memory (4, 22, 55). However, findings varied depending on the cognitive measures used, with global screening tools demonstrating weaker or inconsistent sensitivity to domain-specific impairment.

### 3.3 Overview of studies included in scoping review 2

In scoping review 2, 14 studies were included examining methodological approaches used to assess pain, cognitive performance, and cultural variables.

Of these, 10 studies used self-report pain measures (e.g., VAS questionnaires, categorical scales), 3 used observational or mixed pain assessment tools (e.g., PAINAD, PACSLAC elements), and 1 used a qualitative/unclear structured pain assessment approach. Cognitive assessment methods included 6 studies using MMSE or MoCA (or equivalent global screening tools), 5 used domain-specific neuropsychological tests (e.g., TMT), and 3 had limited or no direct cognitive assessment reported. Some studies employed multiple assessment modalities; therefore, categories are not mutually exclusive.

In relation to cultural considerations, one study explicitly included ethnicity or demographic cultural grouping (39). 13 studies made no explicit reference to cultural variables or analysis.

Further detail on pain assessment methods and their distribution across studies is available in Supplementary Material B.

Across scoping review 2, brief and self-report measures were commonly used, while reporting of culturally informed validation processes was limited. These methodological patterns are considered further in the Discussion.

### 3.4 Methodological characteristics of pain and cognitive assessment

The studies included in this synthesis demonstrate considerable reliance on a small number of widely used cognitive screening tools, particularly the MMSE and MoCA. These measures are frequently used due to practicality and speed of administration, especially in clinical and large-scale research settings. However, across the literature, there is limited consistency in how cognitive outcomes are defined, with many studies relying on global cognitive scores rather than domain-specific assessments.

Some studies employ comprehensive neuropsychological test batteries that assess multiple cognitive domains including attention, working memory, executive functioning, and processing speed. While these approaches provide greater specificity, they are less commonly used due to time demands and the burden they place on participants with cognitive impairment. There is therefore a tendency within the literature to favour simpler screening tools, even when these may not be sensitive enough to detect more subtle or domain-specific cognitive changes associated with pain.

Across studies, there is also limited discussion of how cognitive assessment tools may perform differently across populations, particularly in relation to demographic or cultural differences. In the majority of cases, cognitive measures are applied as standardised tools without consideration of whether they fully capture variation in cognitive performance across diverse groups.

### 3.5 Reporting of cultural variables in included studies

Across the studies in the second review, cultural considerations were largely absent from both the measurement approaches and the interpretation of findings. In many cases, cultural variables were not explicitly measured or reported, and only one study made any reference to differences in participant ethnicity or cultural background in relation to pain or cognitive outcomes (39). Even in these instances, cultural factors were not systematically analysed as part of the study design.

Where demographic information was included, it was typically limited to broad categories (such as non-Hispanic Black and non-Hispanic White participants), without further exploration of how cultural context might influence pain reporting, expression, or cognitive performance. The majority of studies made no reference to cultural or linguistic factors at all, suggesting that these were not considered central to either pain assessment or cognitive evaluation within the included research.

The lack of attention to cultural context extends to the tools and measures used across studies. Standardised cognitive assessments such as the MMSE and the MoCA, along with commonly used pain measures including self-report scales and observational tools, were generally applied without discussion of cultural validity or cross-cultural applicability.

The absence of cultural considerations is not typically acknowledged as a limitation, which suggests that cultural neutrality is often assumed within current assessment practices. Few studies discussed cultural validity or cross-cultural applicability of the assessment tools used. A full breakdown of cultural variables and measurement characteristics across included studies is provided in Supplementary Material B.

## 4 Discussion

### 4.1 Pain, cognition, and dementia: key findings

The current synthesis suggests that chronic pain is associated with poorer cognitive outcomes in populations with dementia and cognitive impairment, particularly in processing speed and executive functioning. This is in line with meta-analytic and cohort evidence reporting similar associations (4, 55, 68, 70). However, a substantial proportion of findings emerge from cross-sectional or observational designs and heterogeneous cognitive measures. This suggests that some observed associations between pain and cognition may also be influenced by similarities in study methodology, particularly the widespread reliance on broad cognitive screening tools, rather than clearly defined domain-specific mechanisms. Additionally, this relationship is not entirely linear and varies based on individual characteristics, dementia subtypes, and specific cognitive domains affected (22, 60). Meta-analytic evidence supports a relationship between chronic pain and cognitive decline at a population level, with meta-analytic evidence indicating a small-to-moderate increased risk of dementia among individuals with chronic pain (10), although substantial heterogeneity across studies suggests that this association is influenced by methodological and clinical factors.

### 4.2 Rethinking bidirectionality: clinical relationship or methodological artifact?

Findings from the current synthesis suggest that the proposed bidirectional relationship between pain and cognition should be interpreted cautiously. The interpretation that the apparent bidirectional relationship should be treated cautiously is supported by the predominance of cross-sectional and observational studies within the literature, which limit causal inference (58–60) The observed inconsistencies may therefore partly reflect methodological variation such as differences in cognitive measures and pain assessment strategies (38, 39), rather than true contradictions in findings. Future longitudinal research using multimodal pain assessment approaches and more sensitive domain-specific cognitive measures may help to clarify the nature and direction of these relationships in individuals living with dementia.

### 4.3 Addressing methodological gaps through multimodal assessment

The synthesis identified a clear methodological tension between feasibility and sensitivity in pain assessment approaches. A key limitation concerns the reliability of self-reported pain measures as cognitive decline progresses. While early-stage dementia patients may still provide reliable self-reports, those in later stages require behavioural assessment tools (35, 38, 71, 72). This creates a methodological contradiction within the field, where the dominant assessment approach becomes least appropriate in the populations most affected. Observational measures are often introduced as alternatives; however, despite their value, they remain vulnerable to misinterpretation of pain-related behaviours and an over-reliance on facial expressions, which may not fully capture an individual's pain experience (34–36, 39). A recent systematic review also identified a limited evidence base evaluating dementia-specific pain assessment tools in emergency department settings (37). Current pain measurement practices may therefore privilege observable behaviour over subjective pain experience, shaping the type of evidence that is generated in this literature.

These findings support a multimodal approach to pain assessment that uses self-report where possible and supplements it with observational and behavioural measures, alongside relevant clinical information and input from caregivers or healthcare professionals (34–36, 39). Assessment approaches should be tailored to the stage and severity of dementia rather than applying a single method across the dementia spectrum. A similar tension between feasibility and sensitivity is evident in the cognitive assessment approaches used across dementia research, particularly in the widespread reliance on global screening tools.

### 4.4 Cognitive assessment: beyond traditional screening tools

The synthesis indicates that commonly used cognitive assessment tools may contribute to important methodological constraints within the evidence base. While comprehensive neuropsychological batteries provide a detailed evaluation of cognitive function in multiple domains, they are often time-consuming and impractical for both research and clinical settings. Similarly, commonly used cognitive screening tools such as the Montreal Cognitive Assessment (MoCA) and Mini Mental State Examination (MMSE) provide broad indices of cognitive functioning but are limited in their sensitivity to domain-specific impairments relevant to pain-related cognitive disruption (38, 39). Their widespread use across studies contributes to a homogenised representation of cognition in dementia research, where nuanced differences in executive functioning, attention, and working memory may be collapsed into global scores, thereby obscuring more specific pain-cognition interactions.

More detailed neuropsychological assessments offer greater specificity but are often impractical in applied or clinical research contexts, which reinforces the continued reliance on global screening tools. This tension suggests that the field is constrained not only by the available methods but also by the pragmatic choices that shape what is considered measurable cognition. Importantly, these methodological assumptions are often treated as universally applicable despite being shaped by culturally specific understandings of cognition and behaviour. This pattern was consistent across scoping review 2.

### 4.5 Cultural influences: bridging a major research gap

Cultural considerations remain largely peripheral within dementia-related pain and cognition research. Findings from the present synthesis indicate that cultural factors were rarely incorporated into the included studies, either as variables within study designs or as considerations in the selection and interpretation of assessment tools. This pattern is consistent with prior evidence showing limited integration of cultural variables in both pain and cognitive assessment studies (39, 48). Across the two reviews, cultural factors are largely absent from empirical and methodological consideration, suggesting that the cultural assumptions underpinning pain and cognitive assessment frameworks are insufficiently examined within dementia research (55, 62). Existing literature indicates that cultural norms and values may influence pain reporting, expression, and management strategies (54, 63). Across scoping review 1, only a small number of studies explicitly considered ethnicity or cultural context (e.g., Tanner et al., (39)), and this pattern was consistent in scoping review 2 where most studies did not report cultural variables.

The limited reporting of cultural variables is not merely a gap in the characteristics measured within individual studies but raises broader questions about how pain expression and cognitive performance are treated as comparable across populations. When assessment tools are applied without consideration of language, education, cultural familiarity, or migration-related factors, differences in reported pain or cognitive performance may be interpreted as clinical differences when they may partly reflect differences in assessment validity or communication. This concern is consistent with evidence highlighting the limited research available on dementia care for Arabic-speaking migrants and refugees and the lack of consensus regarding culturally and linguistically appropriate neurocognitive screening, assessment, and care (43). However, the evidence concerning Arabic-speaking migrant and refugee populations should be understood as illustrating a specific and important example of a broader need for culturally appropriate assessment, rather than being assumed to represent all culturally diverse populations.

These concerns about the cultural validity and comparability of assessment are also relevant to pain assessment, as evidence indicates cultural variation in pain reporting and healthcare communication patterns (40–42) These factors may influence test validity, pain reporting accuracy, and comparability across populations. Research by Taiebine (43) further indicates Arabic-speaking populations may experience additional barriers related to diglossia, translation practices, and literacy differences which can affect the validity of neuropsychological testing and dementia diagnosis. This raises questions about the generalisability of findings across populations and suggests that current evidence may disproportionately reflect culturally specific patterns of reporting rather than universal mechanisms.

### 4.6 Implications for future research

The limitations identified across the literature are not isolated methodological issues but are interconnected. Reliance on global cognitive measures, dependence on self-report in cognitively impaired populations, and lack of culturally informed frameworks collectively contribute to a fragmented evidence base. This lack of cultural integration suggests that culture is not simply missing data but may represent a structural limitation affecting the interpretation of pain-cognition relationships. Methodological literature highlights the influence of assessment choices on observed outcomes in cognitive aging research (38, 39).

Future research should adopt pain assessment strategies that are appropriate to the stage and severity of cognitive impairment. Where individuals retain the capacity to communicate their subjective experience, self-report should remain an important component of assessment. However, as cognitive impairment progresses, self-report should be supplemented with observational and behavioural measures rather than replaced by a single alternative tool. A multimodal approach offers a more comprehensive understanding of pain by combining subjective reports, behavioural observations, clinical information, and input from caregivers or healthcare professionals.

Future studies should also consider whether global cognitive screening measures are sufficient for the research questions being addressed. While tools such as the MMSE and MoCA remain valuable because of their practicality, research examining pain-cognition relationships may benefit from greater use of domain-specific measures targeting executive functioning, attention, working memory, and processing speed. Furthermore, greater consistency in selecting and reporting cognitive outcomes would enhance comparability across studies and help establish whether pain specifically affects certain cognitive domains or simply leads to global cognitive decline.

Cultural and linguistic factors should be incorporated more systematically into future research. Studies should report relevant characteristics such as language background, educational experience, ethnicity, cultural background, and, where relevant, migration history. Researchers should also consider whether the pain and cognitive assessment tools used have been validated for the populations being studied. Translation or interpretation alone should not be assumed to ensure cultural equivalence, particularly where assessment performance may be influenced by language structure, literacy, educational background, or cultural familiarity with test materials.

Finally, longitudinal research using consistent and multimodal assessment approaches is needed to clarify the temporal and potentially bidirectional relationships between pain and cognition. Greater attention should be given to potential confounding factors, including dementia subtype, disease severity, comorbidities, medication use, and demographic and cultural characteristics. Such approaches would help distinguish whether observed associations reflect underlying clinical relationships, methodological differences in measurement, or interactions between both.

### 4.7 Limitations

This synthesis is subject to limitations. Both included scoping reviews were conducted as part of the same doctoral research programme, which may increase the potential overlap in interpretation. Additionally, the included reviews restricted studies to English-language publications within the previous five years, which may have excluded relevant earlier research. As a critical synthesis, the analysis involved interpretative integration, meaning thematic development was influenced by the interpretive perspective of the researcher.

Furthermore, because the synthesis was based on two scoping reviews rather than a formal quantitative meta-analysis, the findings should be interpreted as an integrated critical account of patterns within the literature rather than as estimates of the magnitude or causal direction of pain-cognition relationships.

### 4.8 Conclusion

The intersection of pain, cognition, and cultural factors in dementia and cognitive impairment remains a complex but underdeveloped area of research. Rather than treating inconsistencies in the literature as purely empirical discrepancies, this synthesis argues that they may be partly shaped by methodological choices and culturally bounded measurement frameworks.

Relying on tools that become less suitable as cognitive impairment progresses, alongside the use of broad cognitive screening measures, may contribute to a potentially oversimplified understanding of pain-cognition relationships. Findings across the literature therefore may reflect methodological limitations as much as underlying clinical phenomena.

Overall, this synthesis suggests that progress in this field requires not only improved measurement approaches but also a broader re-examination of how pain and cognition are conceptualised and operationalised in dementia research. Improving understanding of pain-cognition interactions in dementia will require simultaneous reform of methodological practice and cultural assumptions within the field.
