# Supplementary material for: Pain, cognition, and culture in dementia research: a critical synthesis of empirical and methodological literature
Source: Front Pain Res (Lausanne). 2026 Oct 9;7:1797629. doi: 10.3389/fpain.2026.1797629 (PMC13691539; doi:10.3389/fpain.2026.1797629)
Supplement: Supplementary file 1 [file Table1.docx]

**Supplementary File A: Shared Search Strategy Used Across Both Scoping Reviews**

The following search strategy was used across both scoping reviews included in the critical synthesis. Searches were conducted in PubMed and PsycINFO on 29 August 2021 using combinations of terms relating to chronic pain, cognition, dementia, communication, and culture. Searches were restricted to English-language studies and human participants. Across both databases, the search returned 7,013 records in total prior to deduplication

**Table A1.** Shared search strategy used across both scoping reviews

| Search Number | Search terms | Records retrieved |
| --- | --- | --- |
| 1 | ("chronic pain" OR "persistent pain" OR "neuropathic pain") AND ("dementia" OR "Alzheimer’s disease" OR "cognitive decline") | 358 |
| 2 | ("pain communication" OR "pain assessment" OR "nonverbal communication") AND ("dementia" OR "cognitive impairment") | 261 |
| 3 | ("culture" OR "cultural competence" OR "cross-cultural") AND ("dementia" OR "cognitive decline") AND ("pain management") | 4 |
| 4 | ("chronic pain" OR "persistent pain") AND ("dementia" OR "Alzheimer’s disease") AND ("communication" OR "patient-provider communication") AND ("culture" OR "cultural sensitivity") | 0 |

PubMed:

PsychInfo:

| Search Number | Search terms | Records retrieved |
| --- | --- | --- |
| 1 | ("chronic pain" OR "persistent pain" OR "neuropathic pain") AND ("dementia" OR "Alzheimer’s disease" OR "cognitive decline") | 123 |
| 2 | ("pain communication" OR "pain assessment" OR "nonverbal communication") AND ("dementia" OR "cognitive impairment") | 156 |
| 3 | ("culture" OR "cultural competence" OR "cross-cultural") AND ("dementia" OR "cognitive decline") AND ("pain management") | 2 |
| 4 | ("chronic pain" OR "persistent pain") AND ("dementia" OR "Alzheimer’s disease") AND ("communication" OR "patient-provider communication") AND ("culture" OR "cultural sensitivity") | 6,109 |
